# Acridinium (6-carboxy­pyridine-2-carboxyl­ato)(pyridine-2,6-dicarboxyl­ato)zincate(II) penta­hydrate

**DOI:** 10.1107/S1600536809011106

**Published:** 2009-03-31

**Authors:** Masoumeh Tabatabaee, Hossein Aghabozorg, Jafar Attar Gharamaleki, Mahboubeh A. Sharif

**Affiliations:** a Department of Chemistry, Islamic Azad University, Yazd Branch, Yazd, Iran; b Faculty of Chemistry, Tarbiat Moallem University, 49 Mofateh Avenue, Tehran, Iran; c Department of Chemistry, Islamic Azad University, Qom Branch, Qom, Iran

## Abstract

The reaction of Zn(NO_3_)_2_ with pyridine-2,6-dicarboxylic acid (pydcH_2_) and acridine (acr) in aqueous solution leads to the formation of the title compound, (C_13_H_10_N)[Zn(C_7_H_3_NO_4_)(C_7_H_4_NO_4_)]·5H_2_O or (acrH)[Zn(pydcH)(pydc)]·5H_2_O. In the title compound, the Zn^II^ atom is coordinated by four O atoms and two N atoms from the tridentate chelating rings of (pydc)^2−^ and (pydcH)^−^ anions. The geometry of the resulting ZnN_2_O_4_ coordination can be described as distorted octa­hedral. To balance the charges, one protonated acridine (acrH)^+^ cation is present. In the crystal structure, extensive O—H⋯O and N—H⋯O hydrogen bonds involving acrH^+^, the complex anion and uncoordinated water mol­ecules form a three-dimensional network.

## Related literature

For related structures, see: Aghabozorg *et al.* (2009 ▶); Moghimi *et al.* (2005 ▶); Ranjbar *et al.* (2002 ▶); Tabatabaee *et al.* (2008 ▶); Aghabozorg, Attar Gharamaleki *et al.* (2008 ▶); Aghabozorg, Firoozi *et al.* (2008 ▶); Aghabozorg, Manteghi *et al.* (2008 ▶); Safaei-Ghomi et al. (2009 ▶); Soleimannejad *et al.* (2008 ▶).
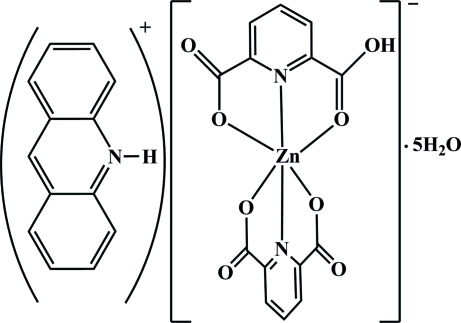


## Experimental

### 

#### Crystal data

(C_13_H_10_N)[Zn(C_7_H_3_NO_4_)(C_7_H_4_NO_4_)]·5H_2_O
*M* *_r_* = 666.89
Monoclinic, 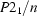

*a* = 9.6083 (5) Å
*b* = 18.9681 (9) Å
*c* = 15.5435 (8) Å
β = 96.051 (1)°
*V* = 2817.0 (2) Å^3^
*Z* = 4
Mo *K*α radiation
μ = 0.95 mm^−1^
*T* = 120 K
0.60 × 0.14 × 0.14 mm

#### Data collection

Bruker SMART 1000 CCD area-detector diffractometer
Absorption correction: multi-scan (*SADABS*; Sheldrick, 1998 ▶) *T*_min_ = 0.652, *T*_max_ = 0.879
26948 measured reflections
7457 independent reflections
5795 reflections with *I* > 2σ(*I*)
*R*_int_ = 0.034

#### Refinement

*R*[*F* ^2^ > 2σ(*F* ^2^)] = 0.041
*wR*(*F* ^2^) = 0.108
*S* = 1.06
7457 reflections
445 parameters
H atoms treated by a mixture of independent and constrained refinement
Δρ_max_ = 0.87 e Å^−3^
Δρ_min_ = −0.35 e Å^−3^

### 

Data collection: *SMART* (Bruker, 1998 ▶); cell refinement: *SAINT-Plus* (Bruker, 1998 ▶); data reduction: *SAINT-Plus*; program(s) used to solve structure: *SHELXTL* (Sheldrick, 2008 ▶); program(s) used to refine structure: *SHELXTL*; molecular graphics: *SHELXTL*; software used to prepare material for publication: *SHELXTL*.

## Supplementary Material

Crystal structure: contains datablocks I, global. DOI: 10.1107/S1600536809011106/bq2129sup1.cif

Structure factors: contains datablocks I. DOI: 10.1107/S1600536809011106/bq2129Isup2.hkl

Additional supplementary materials:  crystallographic information; 3D view; checkCIF report

## Figures and Tables

**Table 1:** Selected bond lengths (Å)

Zn1—N2	2.0011 (16)
Zn1—N1	2.0238 (16)
Zn1—O3	2.0864 (14)
Zn1—O5	2.1443 (15)
Zn1—O7	2.2100 (14)
Zn1—O1	2.3406 (14)

**Table 2:** Hydrogen-bond geometry (Å, °)

*D*—H⋯*A*	*D*—H	H⋯*A*	*D*⋯*A*	*D*—H⋯*A*
O2—H2*O*⋯O3*W*	0.88 (4)	1.61 (4)	2.465 (2)	166 (4)
N3—H3*N*⋯O7	0.83 (3)	1.96 (3)	2.752 (2)	159 (3)
O1*W*—H1*W*1⋯O8	0.79 (3)	1.91 (3)	2.696 (2)	174 (3)
O1*W*—H2*W*1⋯O4^i^	0.85 (4)	2.07 (4)	2.901 (2)	165 (3)
O2*W*—H1*W*2⋯O4	0.82 (3)	2.07 (3)	2.873 (2)	166 (3)
O2*W*—H2*W*2⋯O5*W*^ii^	0.86 (3)	1.98 (3)	2.791 (3)	158 (3)
O3*W*—H1*W*3⋯O1*W*^iii^	0.85 (4)	1.82 (4)	2.665 (2)	173 (3)
O3*W*—H2*W*3⋯O4*W*^iv^	0.85 (4)	1.78 (4)	2.636 (2)	174 (5)
O4*W*—H1*W*4⋯O2*W*^iii^	0.81 (4)	1.95 (4)	2.768 (3)	178 (3)
O4*W*—H2*W*4⋯O5	0.81 (3)	1.98 (3)	2.788 (2)	172 (4)
O5*W*—H1*W*5⋯O6^v^	0.80 (3)	2.08 (3)	2.880 (2)	173 (3)
O5*W*—H2*W*5⋯O6	0.86 (4)	1.99 (5)	2.838 (2)	171 (4)

Symmetry codes: (i) 
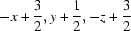
; (ii) 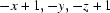; (iii) 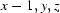; (iv) 
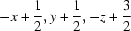
; (v) 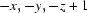.

**Fig. 1.**
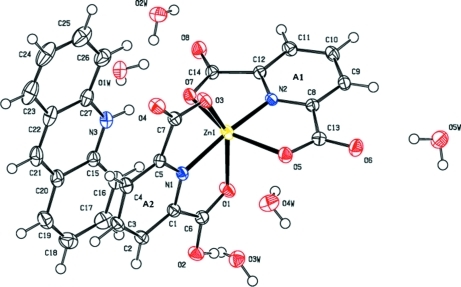
A view of (I), with the atom-numbering scheme and 50% probability displacement ellipsoids.

**Fig. 2.**
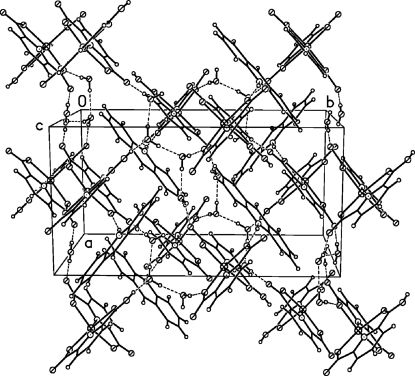
Fragment of crystal packing (projection along c axis). Hydrogen bonds are shown with dashed lines.

**Fig. 3.**
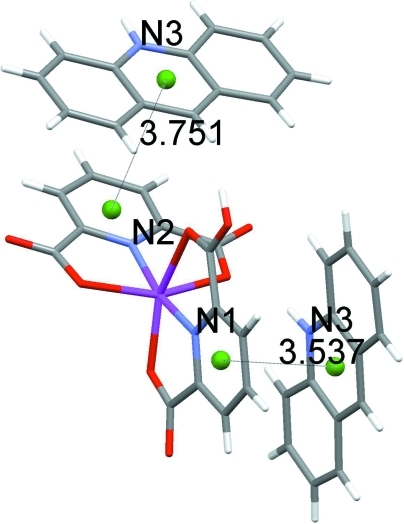
Representation of π-π stacking in (acrH)[Zn(pydcH)(pydc)].5H2O.

## supplementary crystallographic information

### Comment

In the recent years, our research group has been interested in the synthesis of proton transfer compounds and study of their behavior with metal ions. We have focused on the proton delivery from polycarboxylic acids, which are considered as very good donors and amines as acceptors. Among polycarboxylic acids, pyridine-2,6-dicarboxylic acid (pydcH_2_) as a very important carboxylate derivative has attracted much interest in coordination chemistry and it is the one that we utilized widely in our studies (Aghabozorg, Attar Gharamaleki *et al.*, 2008; Aghabozorg, Firoozi *et al.*, 2008; Aghabozorg, Manteghi *et al.*, 2008; Tabatabaee *et al.*, 2008; Soleimannejad *et al.*, 2008; Aghabozorg *et al.*, 2009; Safaei-Ghomi *et al.*, 2009). In order to develop novel systems, we wish to report the first complex of Zn^II^ with pyridine-2,6-dicarboxylic acid as proton donor and acridine as proton acceptor.

The title compound consists of [Zn(pydcH)(pydc)]^- ^ anion, (acrH)^+^ cation and five uncoordinated water molecules (Fig. 1). Zn^II^ ionic complex includes dianionic ((pydc)^2-^) and monoanionic ((pydcH)^–^) forms of pydcH_2_, simultaneously. Zn^II^ atom is six-coordinated by these anions and the geometry of the resulting ZnN2O4 coordination can be described as distorted octahedral (Table 1). The N atoms occupy the axial positions. The N1—Zn1—N2 angle (167.38 (8)°) deviates from linearity. The dihedral angle between the mean planes of the pyridine rings (A1 and A2, defined in Fig. 1) is 88.16 (9)° indicating that (pydc)^2–^ and (pydcH)^– ^ fragments are almost perpendicular to each other. Zn—N distances of (2.0011 (16)Å and 2.0238 (16)Å and Zn—O distances (Zn1—O1:2.0864 (14)Å, Zn1—O5: 2.1443 (15)Å, Zn1—O3:2.2100 (14)Å and Zn1—O7: 2.3406 (14)Å) are consistent with those found in (pydaH)[Zn(pydcH)(pydc)].3H_2_O (Ranjbar *et al.* 2002) and (creatH)[Zn(pydcH)(pydc)].4H_2_O (Moghimi *et al.* 2005). There are some hydrogen bonding interactions such as O–H···O and N–H···O between cations, anions and uncoordinated water molecules (Table 2). The water molecules act also as bridging agents and link anions together *via* hydrogen bonds (Fig. 2). As it is seen in Fig. 3, there are also π-π stacking interactions between the aromatic rings of the coordinated (pydc)^2–^ and (pydcH)^-^ anions and acridinum cation, with distances of 3.537 (1)Å for *Cg*5···*Cg*7 [*Cg*5: N1/C1—C5, *Cg*7:N3/C15—C20—C21—C22—C27] and 3.751 (1)Å for *Cg*6···*Cg*7 (*Cg*6:N2/C8—C12). Ion pairing, hydrogen bonding, π–π stacking and van der Waals interactions are also effective packing for the crystal structure. These interactions lead to formation of a three-dimensional supramolecular structure.

### Experimental

An aqueous solution of zinc nitrate (Zn(NO_3_)_2_. 6H_2_O, (0.15 g, 0.5 mmol) in water (20 ml) was added to a stirring solution of (20 ml) pyridine-2,6-dicarboxylic acid (0.1 g, 0.5 mmol) and 0.25 g (1.5 mmol) acridine. The reaction mixture was stirred at 25°C for 2 h. Colorless crystals of the title compound were obtained after two weeks at room temperature.

### Refinement

The H(C) atoms were positioned geometrically and refined in isotropically in riding model with U_iso_(H)=1.2U_eq_(C). The H atoms of water molecules, OH and NH groups were located in difference Fourier synthesis and refined isotropically.

### Crystal data

**Table:**

(C_13_H_10_N)[Zn(C_7_H_3_NO_4_)(C_7_H_4_NO_4_)]·5H_2_O	*F*(000) = 1376
*M**_r_* = 666.89	*D*_x_ = 1.572 Mg m^−^^3^
Monoclinic, *P*2_1_/*n*	Mo *K*α radiation, λ = 0.71073 Å
Hall symbol: -P 2yn	Cell parameters from 8302 reflections
*a* = 9.6083 (5) Å	θ = 2.2–29.7°
*b* = 18.9681 (9) Å	µ = 0.95 mm^−^^1^
*c* = 15.5435 (8) Å	*T* = 120 K
β = 96.051 (1)°	Prism, yellow
*V* = 2817.0 (2) Å^3^	0.60 × 0.14 × 0.14 mm
*Z* = 4	

### Data collection

**Table:**

Bruker SMART 1000 CCD area-detector diffractometer	7457 independent reflections
Radiation source: normal-focus sealed tube	5795 reflections with *I* > 2σ(*I*)
graphite	*R*_int_ = 0.034
ω scans	θ_max_ = 29.0°, θ_min_ = 1.7°
Absorption correction: multi-scan (*SADABS*; Sheldrick, 1998)	*h* = −13→13
*T*_min_ = 0.652, *T*_max_ = 0.879	*k* = −25→25
26948 measured reflections	*l* = −21→21

### Refinement

**Table:**

Refinement on *F*^2^	Primary atom site location: structure-invariant direct methods
Least-squares matrix: full	Secondary atom site location: difference Fourier map
*R*[*F*^2^ > 2σ(*F*^2^)] = 0.041	Hydrogen site location: inferred from neighbouring sites
*wR*(*F*^2^) = 0.108	H atoms treated by a mixture of independent and constrained refinement
*S* = 1.06	*w* = 1/[σ^2^(*F*_o_^2^) + (0.0451*P*)^2^ + 2.9525*P*] where *P* = (*F*_o_^2^ + 2*F*_c_^2^)/3
7457 reflections	(Δ/σ)_max_ = 0.002
445 parameters	Δρ_max_ = 0.87 e Å^−^^3^
0 restraints	Δρ_min_ = −0.35 e Å^−^^3^

### Special details

**Table:**

Geometry. All e.s.d.'s (except the e.s.d. in the dihedral angle between two l.s. planes) are estimated using the full covariance matrix. The cell e.s.d.'s are taken into account individually in the estimation of e.s.d.'s in distances, angles and torsion angles; correlations between e.s.d.'s in cell parameters are only used when they are defined by crystal symmetry. An approximate (isotropic) treatment of cell e.s.d.'s is used for estimating e.s.d.'s involving l.s. planes.
Refinement. Refinement of *F*^2^ against ALL reflections. The weighted *R*-factor *wR* and goodness of fit *S* are based on *F*^2^, conventional *R*-factors *R* are based on *F*, with *F* set to zero for negative *F*^2^. The threshold expression of *F*^2^ > σ(*F*^2^) is used only for calculating *R*-factors(gt) *etc*. and is not relevant to the choice of reflections for refinement. *R*-factors based on *F*^2^ are statistically about twice as large as those based on *F*, and *R*- factors based on ALL data will be even larger.

### Fractional atomic coordinates and isotropic or equivalent isotropic displacement parameters (Å^2^)

**Table:**

	*x*	*y*	*z*	*U*_iso_*/*U*_eq_	
Zn1	0.49831 (3)	0.093889 (12)	0.701036 (14)	0.01975 (8)	
O1	0.34045 (16)	0.18535 (8)	0.71906 (9)	0.0242 (3)	
O2	0.23932 (16)	0.23960 (8)	0.82468 (10)	0.0231 (3)	
H2O	0.198 (5)	0.261 (2)	0.779 (3)	0.092 (15)*	
O3	0.63925 (16)	0.01596 (8)	0.74853 (9)	0.0227 (3)	
O4	0.73383 (16)	−0.03264 (8)	0.87279 (10)	0.0248 (3)	
O5	0.32802 (16)	0.02475 (8)	0.65903 (9)	0.0229 (3)	
O6	0.20086 (16)	−0.01389 (8)	0.53928 (10)	0.0262 (3)	
O7	0.65542 (15)	0.17569 (8)	0.68086 (9)	0.0218 (3)	
O8	0.74307 (17)	0.23451 (8)	0.57367 (10)	0.0269 (3)	
N1	0.48997 (17)	0.10445 (8)	0.82995 (10)	0.0167 (3)	
N2	0.48458 (17)	0.10381 (8)	0.57225 (10)	0.0155 (3)	
C1	0.4084 (2)	0.15173 (10)	0.86374 (12)	0.0182 (4)	
C2	0.4085 (2)	0.15979 (10)	0.95278 (13)	0.0200 (4)	
H2A	0.3487	0.1928	0.9764	0.024*	
C3	0.5000 (2)	0.11747 (11)	1.00590 (13)	0.0222 (4)	
H3A	0.5039	0.1219	1.0670	0.027*	
C4	0.5854 (2)	0.06882 (11)	0.96989 (13)	0.0207 (4)	
H4A	0.6485	0.0401	1.0057	0.025*	
C5	0.5764 (2)	0.06307 (10)	0.88014 (12)	0.0168 (4)	
C6	0.3236 (2)	0.19449 (10)	0.79562 (13)	0.0191 (4)	
C7	0.6578 (2)	0.01088 (10)	0.83018 (13)	0.0193 (4)	
C8	0.3880 (2)	0.06609 (10)	0.52419 (12)	0.0170 (4)	
C9	0.3753 (2)	0.07039 (11)	0.43455 (12)	0.0198 (4)	
H9A	0.3063	0.0438	0.4003	0.024*	
C10	0.4666 (2)	0.11477 (11)	0.39627 (13)	0.0227 (4)	
H10A	0.4619	0.1176	0.3350	0.027*	
C11	0.5645 (2)	0.15491 (11)	0.44730 (13)	0.0213 (4)	
H11A	0.6256	0.1862	0.4219	0.026*	
C12	0.5702 (2)	0.14777 (10)	0.53652 (12)	0.0167 (4)	
C13	0.2970 (2)	0.02123 (10)	0.57769 (13)	0.0190 (4)	
C14	0.6661 (2)	0.18976 (10)	0.60159 (13)	0.0189 (4)	
N3	0.76898 (19)	0.21857 (9)	0.84229 (12)	0.0208 (3)	
H3N	0.746 (3)	0.2148 (15)	0.7896 (19)	0.037 (8)*	
C15	0.6925 (2)	0.26041 (10)	0.88993 (13)	0.0204 (4)	
C16	0.5844 (2)	0.30294 (11)	0.85003 (15)	0.0255 (4)	
H16A	0.5673	0.3048	0.7887	0.031*	
C17	0.5043 (3)	0.34143 (11)	0.90007 (16)	0.0302 (5)	
H17A	0.4304	0.3695	0.8731	0.036*	
C18	0.5291 (3)	0.34042 (12)	0.99176 (16)	0.0321 (5)	
H18A	0.4708	0.3671	1.0253	0.039*	
C19	0.6361 (3)	0.30137 (13)	1.03178 (15)	0.0311 (5)	
H19A	0.6538	0.3021	1.0931	0.037*	
C20	0.7215 (2)	0.25951 (11)	0.98225 (13)	0.0238 (4)	
C21	0.8285 (2)	0.21652 (12)	1.01903 (14)	0.0284 (5)	
H21A	0.8496	0.2160	1.0801	0.034*	
C22	0.9057 (2)	0.17410 (11)	0.96824 (14)	0.0263 (4)	
C23	1.0150 (3)	0.12781 (14)	1.00362 (18)	0.0382 (6)	
H23A	1.0393	0.1257	1.0644	0.046*	
C24	1.0833 (3)	0.08721 (13)	0.9503 (2)	0.0426 (7)	
H24A	1.1552	0.0565	0.9743	0.051*	
C25	1.0502 (3)	0.08954 (12)	0.8598 (2)	0.0397 (6)	
H25A	1.1008	0.0606	0.8241	0.048*	
C26	0.9464 (2)	0.13276 (12)	0.82196 (17)	0.0309 (5)	
H26A	0.9245	0.1338	0.7609	0.037*	
C27	0.8730 (2)	0.17567 (11)	0.87635 (14)	0.0231 (4)	
O1W	0.84765 (17)	0.32871 (9)	0.69335 (11)	0.0251 (3)	
H1W1	0.815 (3)	0.3033 (16)	0.656 (2)	0.034 (8)*	
H2W1	0.813 (4)	0.369 (2)	0.681 (2)	0.065 (11)*	
O2W	0.96559 (18)	−0.06357 (9)	0.77630 (12)	0.0279 (3)	
H1W2	0.909 (3)	−0.0493 (17)	0.808 (2)	0.045 (9)*	
H2W2	0.940 (3)	−0.0417 (16)	0.729 (2)	0.039 (8)*	
O3W	0.12555 (19)	0.31700 (9)	0.71080 (10)	0.0262 (3)	
H1W3	0.037 (4)	0.3180 (18)	0.708 (2)	0.051 (9)*	
H2W3	0.170 (5)	0.356 (2)	0.710 (3)	0.084 (14)*	
O4W	0.25508 (19)	−0.05822 (9)	0.79542 (12)	0.0302 (4)	
H1W4	0.170 (4)	−0.0602 (18)	0.791 (2)	0.049 (10)*	
H2W4	0.268 (4)	−0.0327 (18)	0.755 (2)	0.050 (9)*	
O5W	0.0430 (2)	−0.01699 (10)	0.37439 (12)	0.0322 (4)	
H1W5	−0.029 (3)	−0.0086 (15)	0.3945 (19)	0.031 (7)*	
H2W5	0.099 (4)	−0.016 (2)	0.421 (3)	0.066 (11)*	

### Atomic displacement parameters (Å^2^)

**Table:**

	*U*^11^	*U*^22^	*U*^33^	*U*^12^	*U*^13^	*U*^23^
Zn1	0.02513 (13)	0.02160 (13)	0.01244 (12)	−0.00038 (9)	0.00163 (9)	−0.00009 (8)
O1	0.0300 (8)	0.0264 (7)	0.0162 (7)	0.0090 (6)	0.0017 (6)	0.0001 (6)
O2	0.0248 (8)	0.0238 (7)	0.0208 (7)	0.0080 (6)	0.0026 (6)	−0.0013 (6)
O3	0.0293 (8)	0.0239 (7)	0.0149 (6)	0.0062 (6)	0.0015 (6)	−0.0021 (5)
O4	0.0279 (8)	0.0236 (7)	0.0227 (7)	0.0082 (6)	0.0012 (6)	0.0023 (6)
O5	0.0251 (7)	0.0262 (7)	0.0172 (7)	−0.0050 (6)	0.0018 (6)	0.0018 (6)
O6	0.0247 (8)	0.0276 (8)	0.0255 (8)	−0.0079 (6)	−0.0008 (6)	0.0004 (6)
O7	0.0266 (8)	0.0233 (7)	0.0152 (6)	−0.0052 (6)	0.0011 (5)	−0.0005 (5)
O8	0.0298 (8)	0.0273 (8)	0.0236 (7)	−0.0114 (6)	0.0030 (6)	−0.0003 (6)
N1	0.0194 (8)	0.0174 (7)	0.0132 (7)	−0.0023 (6)	0.0014 (6)	−0.0003 (6)
N2	0.0166 (8)	0.0164 (7)	0.0136 (7)	0.0014 (6)	0.0018 (6)	−0.0007 (6)
C1	0.0195 (9)	0.0177 (9)	0.0178 (9)	−0.0024 (7)	0.0030 (7)	−0.0010 (7)
C2	0.0231 (10)	0.0189 (9)	0.0187 (9)	−0.0006 (7)	0.0046 (7)	−0.0029 (7)
C3	0.0281 (10)	0.0245 (10)	0.0146 (9)	−0.0029 (8)	0.0050 (8)	−0.0033 (7)
C4	0.0255 (10)	0.0194 (9)	0.0168 (9)	−0.0012 (8)	0.0001 (7)	0.0021 (7)
C5	0.0172 (9)	0.0153 (8)	0.0178 (9)	−0.0021 (7)	0.0005 (7)	−0.0008 (7)
C6	0.0193 (9)	0.0192 (9)	0.0188 (9)	0.0004 (7)	0.0017 (7)	−0.0003 (7)
C7	0.0205 (9)	0.0185 (9)	0.0190 (9)	0.0001 (7)	0.0020 (7)	−0.0004 (7)
C8	0.0181 (9)	0.0151 (8)	0.0178 (9)	0.0025 (7)	0.0014 (7)	−0.0006 (7)
C9	0.0212 (10)	0.0210 (9)	0.0168 (9)	−0.0002 (7)	−0.0007 (7)	−0.0027 (7)
C10	0.0277 (11)	0.0273 (10)	0.0130 (9)	0.0003 (8)	0.0015 (8)	0.0003 (7)
C11	0.0247 (10)	0.0221 (9)	0.0176 (9)	−0.0013 (8)	0.0045 (8)	0.0023 (7)
C12	0.0173 (9)	0.0161 (8)	0.0164 (8)	0.0017 (7)	0.0004 (7)	0.0000 (7)
C13	0.0200 (9)	0.0170 (9)	0.0203 (9)	0.0007 (7)	0.0031 (7)	0.0007 (7)
C14	0.0192 (9)	0.0177 (9)	0.0197 (9)	−0.0009 (7)	0.0014 (7)	−0.0015 (7)
N3	0.0238 (9)	0.0194 (8)	0.0183 (8)	−0.0032 (7)	−0.0024 (7)	0.0010 (6)
C15	0.0220 (10)	0.0191 (9)	0.0197 (9)	−0.0058 (7)	0.0007 (7)	0.0009 (7)
C16	0.0284 (11)	0.0206 (10)	0.0267 (10)	−0.0008 (8)	−0.0011 (8)	0.0020 (8)
C17	0.0298 (12)	0.0193 (10)	0.0418 (13)	−0.0009 (8)	0.0054 (10)	0.0004 (9)
C18	0.0361 (13)	0.0248 (11)	0.0375 (13)	−0.0055 (9)	0.0132 (10)	−0.0072 (9)
C19	0.0388 (13)	0.0325 (12)	0.0233 (10)	−0.0139 (10)	0.0084 (9)	−0.0064 (9)
C20	0.0277 (11)	0.0225 (10)	0.0206 (10)	−0.0095 (8)	0.0006 (8)	0.0002 (8)
C21	0.0327 (12)	0.0317 (11)	0.0193 (10)	−0.0119 (9)	−0.0049 (9)	0.0051 (8)
C22	0.0240 (10)	0.0238 (10)	0.0289 (11)	−0.0070 (8)	−0.0081 (8)	0.0083 (8)
C23	0.0312 (13)	0.0341 (13)	0.0452 (14)	−0.0058 (10)	−0.0146 (11)	0.0147 (11)
C24	0.0259 (12)	0.0279 (12)	0.070 (2)	0.0008 (10)	−0.0132 (12)	0.0085 (12)
C25	0.0268 (12)	0.0219 (11)	0.0692 (19)	−0.0018 (9)	0.0003 (12)	−0.0086 (11)
C26	0.0247 (11)	0.0251 (11)	0.0424 (13)	−0.0023 (9)	0.0014 (10)	−0.0060 (9)
C27	0.0213 (10)	0.0190 (9)	0.0280 (10)	−0.0059 (8)	−0.0028 (8)	0.0014 (8)
O1W	0.0268 (8)	0.0240 (8)	0.0233 (8)	−0.0020 (6)	−0.0036 (6)	−0.0009 (6)
O2W	0.0274 (8)	0.0299 (8)	0.0273 (8)	0.0023 (7)	0.0068 (7)	−0.0007 (7)
O3W	0.0239 (8)	0.0250 (8)	0.0290 (8)	0.0035 (6)	−0.0010 (6)	−0.0002 (6)
O4W	0.0274 (9)	0.0298 (9)	0.0341 (9)	0.0025 (7)	0.0064 (7)	0.0095 (7)
O5W	0.0264 (9)	0.0412 (10)	0.0288 (9)	−0.0026 (7)	0.0019 (7)	0.0048 (7)

### Geometric parameters (Å, °)

**Table:**

Zn1—N2	2.0011 (16)	N3—C27	1.352 (3)
Zn1—N1	2.0238 (16)	N3—C15	1.354 (3)
Zn1—O3	2.0864 (14)	N3—H3N	0.83 (3)
Zn1—O5	2.1443 (15)	C15—C16	1.407 (3)
Zn1—O7	2.2100 (14)	C15—C20	1.433 (3)
Zn1—O1	2.3406 (14)	C16—C17	1.362 (3)
O1—C6	1.230 (2)	C16—H16A	0.9500
O2—C6	1.292 (2)	C17—C18	1.420 (4)
O2—H2O	0.87 (5)	C17—H17A	0.9500
O3—C7	1.267 (2)	C18—C19	1.363 (4)
O4—C7	1.246 (2)	C18—H18A	0.9500
O5—C13	1.270 (2)	C19—C20	1.425 (3)
O6—C13	1.240 (2)	C19—H19A	0.9500
O7—C14	1.275 (2)	C20—C21	1.387 (3)
O8—C14	1.234 (2)	C21—C22	1.395 (3)
N1—C5	1.334 (2)	C21—H21A	0.9500
N1—C1	1.335 (2)	C22—C27	1.430 (3)
N2—C12	1.333 (2)	C22—C23	1.433 (3)
N2—C8	1.336 (2)	C23—C24	1.351 (4)
C1—C2	1.392 (3)	C23—H23A	0.9500
C1—C6	1.503 (3)	C24—C25	1.411 (4)
C2—C3	1.395 (3)	C24—H24A	0.9500
C2—H2A	0.9500	C25—C26	1.374 (4)
C3—C4	1.391 (3)	C25—H25A	0.9500
C3—H3A	0.9500	C26—C27	1.414 (3)
C4—C5	1.393 (3)	C26—H26A	0.9500
C4—H4A	0.9500	O1W—H1W1	0.79 (3)
C5—C7	1.524 (3)	O1W—H2W1	0.84 (4)
C8—C9	1.388 (3)	O2W—H1W2	0.82 (3)
C8—C13	1.527 (3)	O2W—H2W2	0.85 (3)
C9—C10	1.394 (3)	O3W—H1W3	0.85 (4)
C9—H9A	0.9500	O3W—H2W3	0.85 (5)
C10—C11	1.392 (3)	O4W—H1W4	0.82 (4)
C10—H10A	0.9500	O4W—H2W4	0.81 (4)
C11—C12	1.389 (3)	O5W—H1W5	0.80 (3)
C11—H11A	0.9500	O5W—H2W5	0.86 (4)
C12—C14	1.520 (3)		
			
N2—Zn1—N1	167.38 (7)	C10—C11—H11A	121.0
N2—Zn1—O3	113.11 (6)	N2—C12—C11	121.08 (18)
N1—Zn1—O3	79.15 (6)	N2—C12—C14	114.08 (16)
N2—Zn1—O5	77.60 (6)	C11—C12—C14	124.79 (18)
N1—Zn1—O5	104.68 (6)	O6—C13—O5	126.33 (19)
O3—Zn1—O5	96.92 (6)	O6—C13—C8	118.50 (17)
N2—Zn1—O7	76.47 (6)	O5—C13—C8	115.16 (17)
N1—Zn1—O7	99.78 (6)	O8—C14—O7	126.56 (18)
O3—Zn1—O7	96.89 (6)	O8—C14—C12	118.05 (17)
O5—Zn1—O7	153.75 (5)	O7—C14—C12	115.37 (17)
N2—Zn1—O1	94.29 (6)	C27—N3—C15	124.05 (19)
N1—Zn1—O1	73.40 (6)	C27—N3—H3N	116 (2)
O3—Zn1—O1	152.55 (5)	C15—N3—H3N	119 (2)
O5—Zn1—O1	90.37 (6)	N3—C15—C16	120.89 (19)
O7—Zn1—O1	87.57 (6)	N3—C15—C20	118.76 (19)
C6—O1—Zn1	111.93 (13)	C16—C15—C20	120.3 (2)
C6—O2—H2O	106 (3)	C17—C16—C15	119.4 (2)
C7—O3—Zn1	115.21 (12)	C17—C16—H16A	120.3
C13—O5—Zn1	115.26 (12)	C15—C16—H16A	120.3
C14—O7—Zn1	114.18 (12)	C16—C17—C18	121.4 (2)
C5—N1—C1	121.40 (17)	C16—C17—H17A	119.3
C5—N1—Zn1	115.70 (13)	C18—C17—H17A	119.3
C1—N1—Zn1	122.84 (13)	C19—C18—C17	120.3 (2)
C12—N2—C8	121.65 (16)	C19—C18—H18A	119.9
C12—N2—Zn1	119.81 (13)	C17—C18—H18A	119.9
C8—N2—Zn1	118.53 (13)	C18—C19—C20	120.4 (2)
N1—C1—C2	121.71 (18)	C18—C19—H19A	119.8
N1—C1—C6	112.49 (16)	C20—C19—H19A	119.8
C2—C1—C6	125.77 (18)	C21—C20—C19	123.3 (2)
C1—C2—C3	117.39 (18)	C21—C20—C15	118.5 (2)
C1—C2—H2A	121.3	C19—C20—C15	118.2 (2)
C3—C2—H2A	121.3	C20—C21—C22	121.4 (2)
C4—C3—C2	120.34 (18)	C20—C21—H21A	119.3
C4—C3—H3A	119.8	C22—C21—H21A	119.3
C2—C3—H3A	119.8	C21—C22—C27	118.6 (2)
C3—C4—C5	118.55 (18)	C21—C22—C23	123.2 (2)
C3—C4—H4A	120.7	C27—C22—C23	118.1 (2)
C5—C4—H4A	120.7	C24—C23—C22	119.9 (2)
N1—C5—C4	120.58 (18)	C24—C23—H23A	120.1
N1—C5—C7	113.94 (16)	C22—C23—H23A	120.1
C4—C5—C7	125.47 (18)	C23—C24—C25	121.4 (2)
O1—C6—O2	125.66 (19)	C23—C24—H24A	119.3
O1—C6—C1	119.23 (18)	C25—C24—H24A	119.3
O2—C6—C1	115.09 (17)	C26—C25—C24	121.5 (3)
O4—C7—O3	126.57 (19)	C26—C25—H25A	119.2
O4—C7—C5	117.59 (17)	C24—C25—H25A	119.2
O3—C7—C5	115.84 (17)	C25—C26—C27	118.2 (2)
N2—C8—C9	120.81 (18)	C25—C26—H26A	120.9
N2—C8—C13	113.42 (16)	C27—C26—H26A	120.9
C9—C8—C13	125.76 (18)	N3—C27—C26	120.4 (2)
C8—C9—C10	118.12 (18)	N3—C27—C22	118.6 (2)
C8—C9—H9A	120.9	C26—C27—C22	120.9 (2)
C10—C9—H9A	120.9	H1W1—O1W—H2W1	105 (3)
C11—C10—C9	120.35 (18)	H1W2—O2W—H2W2	102 (3)
C11—C10—H10A	119.8	H1W3—O3W—H2W3	119 (4)
C9—C10—H10A	119.8	H1W4—O4W—H2W4	101 (3)
C12—C11—C10	117.95 (19)	H1W5—O5W—H2W5	99 (3)
C12—C11—H11A	121.0		
			
N2—Zn1—O1—C6	−179.71 (14)	Zn1—O3—C7—C5	−1.0 (2)
N1—Zn1—O1—C6	−2.52 (14)	N1—C5—C7—O4	−175.43 (18)
O3—Zn1—O1—C6	−3.2 (2)	C4—C5—C7—O4	3.6 (3)
O5—Zn1—O1—C6	102.70 (14)	N1—C5—C7—O3	3.8 (3)
O7—Zn1—O1—C6	−103.48 (14)	C4—C5—C7—O3	−177.18 (19)
N2—Zn1—O3—C7	175.78 (14)	C12—N2—C8—C9	1.3 (3)
N1—Zn1—O3—C7	−1.10 (14)	Zn1—N2—C8—C9	−179.21 (14)
O5—Zn1—O3—C7	−104.79 (15)	C12—N2—C8—C13	−177.72 (16)
O7—Zn1—O3—C7	97.59 (14)	Zn1—N2—C8—C13	1.8 (2)
O1—Zn1—O3—C7	−0.5 (2)	N2—C8—C9—C10	0.4 (3)
N2—Zn1—O5—C13	0.03 (14)	C13—C8—C9—C10	179.29 (18)
N1—Zn1—O5—C13	167.26 (14)	C8—C9—C10—C11	−1.8 (3)
O3—Zn1—O5—C13	−112.19 (14)	C9—C10—C11—C12	1.6 (3)
O7—Zn1—O5—C13	9.1 (2)	C8—N2—C12—C11	−1.5 (3)
O1—Zn1—O5—C13	94.34 (14)	Zn1—N2—C12—C11	178.99 (14)
N2—Zn1—O7—C14	−1.10 (13)	C8—N2—C12—C14	176.01 (16)
N1—Zn1—O7—C14	−168.79 (14)	Zn1—N2—C12—C14	−3.5 (2)
O3—Zn1—O7—C14	111.08 (14)	C10—C11—C12—N2	0.0 (3)
O5—Zn1—O7—C14	−10.2 (2)	C10—C11—C12—C14	−177.22 (19)
O1—Zn1—O7—C14	−96.10 (14)	Zn1—O5—C13—O6	−178.26 (17)
N2—Zn1—N1—C5	−163.5 (3)	Zn1—O5—C13—C8	0.8 (2)
O3—Zn1—N1—C5	3.30 (13)	N2—C8—C13—O6	177.49 (17)
O5—Zn1—N1—C5	97.69 (14)	C9—C8—C13—O6	−1.5 (3)
O7—Zn1—N1—C5	−91.92 (14)	N2—C8—C13—O5	−1.7 (2)
O1—Zn1—N1—C5	−176.39 (15)	C9—C8—C13—O5	179.34 (19)
N2—Zn1—N1—C1	13.6 (4)	Zn1—O7—C14—O8	177.92 (17)
O3—Zn1—N1—C1	−179.60 (16)	Zn1—O7—C14—C12	−0.3 (2)
O5—Zn1—N1—C1	−85.21 (15)	N2—C12—C14—O8	−176.04 (18)
O7—Zn1—N1—C1	85.18 (15)	C11—C12—C14—O8	1.4 (3)
O1—Zn1—N1—C1	0.71 (14)	N2—C12—C14—O7	2.3 (2)
N1—Zn1—N2—C12	76.6 (3)	C11—C12—C14—O7	179.78 (19)
O3—Zn1—N2—C12	−89.25 (15)	C27—N3—C15—C16	−179.05 (19)
O5—Zn1—N2—C12	178.44 (15)	C27—N3—C15—C20	−0.4 (3)
O7—Zn1—N2—C12	2.55 (14)	N3—C15—C16—C17	176.4 (2)
O1—Zn1—N2—C12	89.01 (14)	C20—C15—C16—C17	−2.2 (3)
N1—Zn1—N2—C8	−102.9 (3)	C15—C16—C17—C18	1.0 (3)
O3—Zn1—N2—C8	91.25 (14)	C16—C17—C18—C19	1.1 (3)
O5—Zn1—N2—C8	−1.06 (13)	C17—C18—C19—C20	−1.9 (3)
O7—Zn1—N2—C8	−176.94 (15)	C18—C19—C20—C21	−177.4 (2)
O1—Zn1—N2—C8	−90.49 (14)	C18—C19—C20—C15	0.7 (3)
C5—N1—C1—C2	−0.5 (3)	N3—C15—C20—C21	1.0 (3)
Zn1—N1—C1—C2	−177.47 (14)	C16—C15—C20—C21	179.63 (19)
C5—N1—C1—C6	177.80 (17)	N3—C15—C20—C19	−177.22 (18)
Zn1—N1—C1—C6	0.9 (2)	C16—C15—C20—C19	1.4 (3)
N1—C1—C2—C3	1.4 (3)	C19—C20—C21—C22	177.2 (2)
C6—C1—C2—C3	−176.73 (19)	C15—C20—C21—C22	−0.9 (3)
C1—C2—C3—C4	−0.8 (3)	C20—C21—C22—C27	0.2 (3)
C2—C3—C4—C5	−0.6 (3)	C20—C21—C22—C23	−178.6 (2)
C1—N1—C5—C4	−0.9 (3)	C21—C22—C23—C24	178.6 (2)
Zn1—N1—C5—C4	176.22 (14)	C27—C22—C23—C24	−0.2 (3)
C1—N1—C5—C7	178.16 (17)	C22—C23—C24—C25	0.4 (4)
Zn1—N1—C5—C7	−4.7 (2)	C23—C24—C25—C26	−0.5 (4)
C3—C4—C5—N1	1.5 (3)	C24—C25—C26—C27	0.3 (4)
C3—C4—C5—C7	−177.51 (19)	C15—N3—C27—C26	178.98 (19)
Zn1—O1—C6—O2	−177.61 (16)	C15—N3—C27—C22	−0.3 (3)
Zn1—O1—C6—C1	3.8 (2)	C25—C26—C27—N3	−179.3 (2)
N1—C1—C6—O1	−3.4 (3)	C25—C26—C27—C22	−0.1 (3)
C2—C1—C6—O1	174.9 (2)	C21—C22—C27—N3	0.4 (3)
N1—C1—C6—O2	177.91 (17)	C23—C22—C27—N3	179.27 (19)
C2—C1—C6—O2	−3.8 (3)	C21—C22—C27—C26	−178.9 (2)
Zn1—O3—C7—O4	178.12 (17)	C23—C22—C27—C26	0.0 (3)

### Hydrogen-bond geometry (Å, °)

**Table:**

*D*—H···*A*	*D*—H	H···*A*	*D*···*A*	*D*—H···*A*
O2—H2O···O3W	0.88 (4)	1.61 (4)	2.465 (2)	166 (4)
N3—H3N···O7	0.83 (3)	1.96 (3)	2.752 (2)	159 (3)
O1W—H1W1···O8	0.79 (3)	1.91 (3)	2.696 (2)	174 (3)
O1W—H2W1···O4^i^	0.85 (4)	2.07 (4)	2.901 (2)	165 (3)
O2W—H1W2···O4	0.82 (3)	2.07 (3)	2.873 (2)	166 (3)
O2W—H2W2···O5W^ii^	0.86 (3)	1.98 (3)	2.791 (3)	158 (3)
O3W—H1W3···O1W^iii^	0.85 (4)	1.82 (4)	2.665 (2)	173 (3)
O3W—H2W3···O4W^iv^	0.85 (4)	1.78 (4)	2.636 (2)	174 (5)
O4W—H1W4···O2W^iii^	0.81 (4)	1.95 (4)	2.768 (3)	178 (3)
O4W—H2W4···O5	0.81 (3)	1.98 (3)	2.788 (2)	172 (4)
O5W—H1W5···O6^v^	0.80 (3)	2.08 (3)	2.880 (2)	173 (3)
O5W—H2W5···O6	0.86 (4)	1.99 (5)	2.838 (2)	171 (4)

Symmetry codes: (i) −*x*+3/2, *y*+1/2, −*z*+3/2; (ii) −*x*+1, −*y*, −*z*+1; (iii) *x*−1, *y*, *z*; (iv) −*x*+1/2, *y*+1/2, −*z*+3/2; (v) −*x*, −*y*, −*z*+1.
